# Exogenous melatonin strongly affects dynamic photosynthesis and enhances water-water cycle in tobacco

**DOI:** 10.3389/fpls.2022.917784

**Published:** 2022-08-03

**Authors:** Hu Sun, Xiao-Qian Wang, Zhi-Lan Zeng, Ying-Jie Yang, Wei Huang

**Affiliations:** ^1^Kunming Institute of Botany, Chinese Academy of Sciences, Kunming, China; ^2^University of Chinese Academy of Sciences, Beijing, China; ^3^School of Life Sciences, Northwest University, Xi’an, China

**Keywords:** melatonin, photosynthesis, fluctuating light, stomatal conductance, mesophyll conductance, photoprotection

## Abstract

Melatonin (MT), an important phytohormone synthesized naturally, was recently used to improve plant resistance against abiotic and biotic stresses. However, the effects of exogenous melatonin on photosynthetic performances have not yet been well clarified. We found that spraying of exogenous melatonin (100 μM) to leaves slightly affected the steady state values of CO_2_ assimilation rate (*A*_*N*_), stomatal conductance (*g*_*s*_) and mesophyll conductance (*g*_*m*_) under high light in tobacco leaves. However, this exogenous melatonin strongly delayed the induction kinetics of *g*_*s*_ and *g*_*m*_, leading to the slower induction speed of *A*_*N*_. During photosynthetic induction, *A*_*N*_ is mainly limited by biochemistry in the absence of exogenous melatonin, but by CO_2_ diffusion conductance in the presence of exogenous melatonin. Therefore, exogenous melatonin can aggravate photosynthetic carbon loss during photosynthetic induction and should be used with care for crop plants grown under natural fluctuating light. Within the first 10 min after transition from low to high light, photosynthetic electron transport rates (ETR) for *A*_*N*_ and photorespiration were suppressed in the presence of exogenous melatonin. Meanwhile, an important alternative electron sink, namely water-water cycle, was enhanced to dissipate excess light energy. These results indicate that exogenous melatonin upregulates water-water cycle to facilitate photoprotection. Taking together, this study is the first to demonstrate that exogenous melatonin inhibits dynamic photosynthesis and improves photoprotection in higher plants.

## Introduction

Melatonin (MT) is an important hormone synthesized naturally in both plants and animals. Many recent studies have documented that MT is critical in several metabolic processes, including ROS scavenging systems (Siddiqui et al., 2020a,b), secondary metabolism (Farouk and Al-Amri, 2019; Jahan et al., 2020), and modulation of nitrogen metabolism (Qiao et al., 2019; Chen et al., 2021; Meng et al., 2021; Kaya et al., 2022). Therefore, MT plays a significant role in plants to cope with biotic and abiotic stresses (Arnao and Hernández-Ruiz, 2015, 2019, 2020). For example, MT promotes plant growth under harsh environmental conditions such as pollution of harmful elements (Farouk and Al-Amri, 2019; Kaya et al., 2019, 2022; Ahammed et al., 2020; Jahan et al., 2020; Seleiman et al., 2020; Hoque et al., 2021; Li S. et al., 2021; Bhat et al., 2022), heat (Ahammed et al., 2018; Jahan et al., 2019), low temperature (Bajwa et al., 2014; Li et al., 2018; Zhang et al., 2021), salinity (Liang et al., 2015; Qi et al., 2020; Siddiqui et al., 2020a), drought (Sharma and Zheng, 2019; Dai et al., 2020; Imran et al., 2021), high light (Ding et al., 2018; Lee and Back, 2018), ultraviolet radiation (Yao et al., 2021), and herbicides (Park et al., 2013; Giraldo Acosta et al., 2022). Therefore, MT is a plant master regulator with great potential for increasing crop yield in agriculture (Wang et al., 2018; Arnao and Hernández-Ruiz, 2019; Bose and Howlader, 2020). Spraying of melatonin to leaves with a moderate concentration of 100 μM was usually used in previous studies, and the photosynthetic capacity was hardly affected by the spraying of MT (Jahan et al., 2020; Kaya et al., 2022). Naturally, plant growth is not only determined by the photosynthetic capacity but also can be affected by the dynamic photosynthesis under fluctuating light (Adachi et al., 2019; Kimura et al., 2020; Yamori et al., 2020). In nature, fluctuating light can affect plant growth by restricting photosynthesis. However, it is unclear whether the spraying of MT can affect the dynamic photosynthesis in healthy leaves. If the spraying of MT improves photosynthetic induction in crops, it can be used as a potential growth promoter. However, if the dynamic photosynthesis in higher plants is inhibited by the spraying of MT, MT should be used with care to avoid environmental pollution.

Under high light, stomatal conductance (*g*_*s*_) and mesophyll conductance (*g*_*m*_) are elevated to increase CO_2_ diffusion from air to the sites of Rubisco carboxylation in chloroplasts and thus contribute to the high level of net CO_2_ assimilation rate (*A*_*N*_) (Oguchi et al., 2003; Xiong et al., 2015, 2018; Ferroni et al., 2021). Under low light, relative low levels of *g*_*s*_ and/or *g*_*m*_ can satisfy the low *A*_*N*_ (Xiong et al., 2018; Qiao et al., 2020; Zhang et al., 2020). Most crop plants cultivated under natural field conditions usually experience dramatic fluctuations of illumination (Pearcy, 1990; Slattery et al., 2018). When light intensity increased abruptly, the low *g*_*s*_ and/or *g*_*m*_ restricted CO_2_ diffusion rate and thus made *A*_*N*_ to be limited by the low chloroplast CO_2_ concentration (De Souza et al., 2020; Liu et al., 2022; Sun et al., 2022). Improved stomatal opening or increased *g*_*s*_ could significantly accelerate the response speed of *A*_*N*_ and thus enhance biomass production in fluctuating light (Kimura et al., 2020; Yamori et al., 2020). Under salinity or nitrogen deficiency conditions, the decreased induction speeds of *g*_*s*_ and *g*_*m*_ restricted *A*_*N*_ during photosynthetic induction, leading to the decline of biomass production under fluctuating light (Zhang et al., 2020; Sun et al., 2022). Therefore, if MT increases the induction speeds of *g*_*s*_ and *g*_*m*_, it can be used as a growth promotor for crop plants under natural fluctuating light. In the other hand, if MT decreases the response kinetics of *g*_*s*_ and *g*_*m*_ under fluctuating light, MT should be used with care to prevent negative effect on plant growth. Therefore, it is necessary to clarify the effects of MT on dynamic changes in *g*_*s*_ and *g*_*m*_.

When CO_2_ assimilation is restricted under environmental stresses, the excess light energy should be finely dissipated harmlessly to avoid photodamage to photosystem I and II (PSI and PSII). For example, fluctuating light causes selective photoinhibition of PSI in angiosperms (Kono et al., 2014; Yamamoto et al., 2016; Huang et al., 2019a; Yamamoto and Shikanai, 2019). When light intensity abruptly increases, electron transport from PSII immediately increases (Sun et al., 2020b; Tan et al., 2021). This rapid change in PSII electron flow is accompanied by much slower kinetics of *A*_*N*_ (Yamamoto et al., 2016). The resulting PSI over-reduction produces reactive oxygen species within PSI and thus causes PSI photoinhibition (Yamamoto and Shikanai, 2019). Owing to the key role of PSI in regulation of photosynthetic electron flow, PSI photoinhibition strongly suppresses *A*_*N*_, photoprotection and plant growth (Sejima et al., 2014; Brestic et al., 2015; Zivcak et al., 2015; Lima-Melo et al., 2019; Shimakawa and Miyake, 2019). Under high light, the inhibition of *A*_*N*_ increases the electron transfer from PSI to oxygen, resulting in the production of reactive oxygen species in chloroplast stroma (Takahashi and Murata, 2005, 2006). Reactive oxygen species inhibit the *de novo* synthesis of PSII proteins, primarily the D1 protein at the translation elongation step in *psbA* expression (Nishiyama et al., 2001, 2005). Under such conditions, the higher rate of PSII photodamage relative to PSII repair accelerates PSII photoinhibition (Murata et al., 2007). If moderate PSII photoinhibition occurred, the oxidation of water at PSII and linear electron flow would be suppressed, restricting regeneration of ATP and NADPH and thus impairing *A*_*N*_ and plant growth (Takahashi and Murata, 2008; Huang et al., 2018; Kaya et al., 2022).

Plants have several photoprotective mechanisms to deal with environmental stress (Takahashi and Badger, 2011; Allahverdiyeva et al., 2015; Shikanai and Yamamoto, 2017; Alboresi et al., 2019). In angiosperms, cyclic electron flow plays the key role in protecting PSI and PSII under excess light (Munekage et al., 2002, 2008; Takahashi et al., 2009; Suorsa et al., 2012; Yamamoto and Shikanai, 2019). In addition, water-water cycle can significantly prevent PSI photoinhibition under fluctuating light (Huang et al., 2019b; Sun et al., 2020a; Yang et al., 2020) and protect PSII under high light (Asada, 1999, 2000; Hirotsu et al., 2004; Yi et al., 2014; Huang et al., 2016). During water-water cycle, electrons splitting from water are transported through photosynthetic electron transport chains and ultimately to oxygen. The resulting reactive oxygen species are converted into water by superoxide dismutase (SOD) and ascorbate peroxidase (APX). The operation of water-water cycle can dissipate excess light energy, increase ΔpH formation and balance ATP/NADPH production ratio (Miyake, 2010; Shikanai and Yamamoto, 2017). Consequently, water-water cycle favors photosynthetic regulation when CO_2_ assimilation is restricted under harsh environmental conditions. As reported in previous studies, exogenous MT can increase the expression of SOD and APX in leaves of higher plants (Kaya et al., 2019; Jahan et al., 2020; Li X. et al., 2021). Because SOD and APX are the two key enzymes in charge of water-water cycle (Asada, 2000), the positive effect of exogenous MT on plant growth under environmental stresses might be related to the enhancement of water-water cycle. However, no study has investigated the effect of exogenous MT on the capacity of water-water cycle.

In the present study, we studied the effect of exogenous MT on dynamic photosynthetic performances in leaves of tobacco. The aims were to (1) understand whether exogenous MT is beneficial or detrimental to dynamic photosynthesis; and (2) explore whether exogenous MT enhances the capacity of water-water cycle. We found that spraying of exogenous MT strongly inhibited the dynamic photosynthesis in healthy leaves of tobacco, suggesting that abuse of MT can restrict the photosynthetic carbon gain under natural fluctuating light. Furthermore, exogenous MT upregulated water-water cycle to favor photoprotection especially when CO_2_ assimilation was restricted.

## Materials and methods

### Plant materials and treatments

Tobacco (*Nicotiana tabacum* cv. K326) plants were grown in an open field with full sunlight. Plants were grown in 19-cm plastic pots with humus soil (the initial soil nitrogen content was 2.1 mg/g). Plants were fertilized with Peters Professional’s water solution (0.15 g N/plant every 2 days) and were watered every day to prevent any nutrient or water stress. After cultivation for 1 month, melatonin solution (MT, 100 μM) or water were sprayed to youngest fully developed leaves. This MT concentration was chosen based on previous studies (Kaya et al., 2019, 2022; Jahan et al., 2020). After spraying twice with the interval of 3 days, photosynthetic measurements were conducted. During the period of treatment, the day/night air temperatures were approximately 30/20 C, the relative air humidity was approximately 60–70%, and the maximum light intensity exposed to leaves was approximately 2,000 μmol photons m^–2^ s^–1^.

### Gas exchange and chlorophyll fluorescence measurements

Gas exchange and chlorophyll fluorescence were measured using a LI-6400XT coupled with a fluorometer (Li-6400-40; Li-Cor Inc., Lincoln, NE, United States). For all measurements, air temperature was approximately 25°C and the vapor pressure deficit was approximately 1.3 kPa. The flow rate within the chamber was set at 300 mmol air min^–1^. After pre-illumination at high light (1,500 μmol photons m^–2^ s^–1^, 90–10% red-blue light) and 400 μmol CO_2_ mol^–1^ air to reach steady-state photosynthesis, leaves were exposed to low light (50 μmol photons m^–2^ s^–1^, 90–10% red-blue light) for 5 min to simulate natural shadefleck. Afterward, photosynthetic induction phases were conducted again at high light (1,500 μmol photons m^–2^ s^–1^), and the steady-state conditions were achieved after 30 min illumination.

During photosynthesis induction, the steady-state fluorescence (*F*_*s*_) and the maximum fluorescence (*F*_*m*_’) were measured for further analysis. *F*_*m*_′ was measured by application of a saturating white light flash of 8,000 μmol m^–2^ s^–1^, and the quantum efficiency of photosystem II (Φ_*PSII*_) was calculated as follows (Genty et al., 1989):


ΦPSII=(Fm-′Fs)Fm′

The electron transport rate (ETR) through PSII was calculated as


$$\text{ETR}=\Phi_{\text{PSII}}\times \text{PPFD}\times \alpha \times \beta$$

where the PPFD value corresponded to the light intensity stated above, the typical value 0.45 was assumed for the product of α × β (Kaiser et al., 2017).

### Estimation of mesophyll conductance, chloroplast CO_2_ concentration, and maximum velocity of rubisco for carboxylation

Based on the combination of gas exchange and ETR, *g*_*m*_ is calculated (Harley et al., 1992):


$${\text{g}}_{\text{m}}=\frac{A_{\text{N}}}{C_{\text{i}}-\Gamma^{*}\,\left(\text{ETR}+8\,\left(A_{\text{N}}+R_{\text{d}}\right)\right)/\left(\text{ETR}-4\,\left(A_{\text{N}}+R_{\text{d}}\right)\right)}$$

where *A*_*N*_ represents the area-based net CO_2_ assimilation rate and Γ* represents the CO_2_ compensation point in the absence of respiration (Farquhar et al., 1980; von Caemmerer and Evans, 2015). The average Γ* for C3 species at 25°C, 41.2 μmol/mol (Hermida-Carrera et al., 2016), was used in this study. In the current study, the day respiration rate (*R*_*d*_) was calculated as half of the dark respiration rate as measured after dark adaptation for 10 min (Carriquí et al., 2015).

Based on the estimated *g*_*m*_, the chloroplast CO_2_ concentration (*C*_*c*_) was calculated (Long and Bernacchi, 2003; Warren and Dreyer, 2006):


$$C_{\text{c}}=C_{\text{i}}-\frac{A_{\text{N}}}{{\text{g}}_{\text{m}}}$$

The maximum velocity of Rubisco for carboxylation (*V*_*cmax*_) at steady-state conditions was calculated with following equation (Farquhar et al., 1980; Eyland et al., 2021):


$$V_{\text{cmax}}\,\frac{\left(A_{\text{N}}+R_{\text{d}}\right)\,\left(C_{\text{i}}+K_{\text{m}}\right)}{\left(C_{\text{i}}-\Gamma^{*}\right)}$$

where *K*_*m*_ is the effective the Rubisco Michaelis–Menten constant for CO_2_ under 21% O_2_, and the average value for C3 species at 25°C, 529.4 μmol mol^–1^ (Hermida-Carrera et al., 2016; Eyland et al., 2021), was used in this study.

### Quantitative limitation analysis of assimilation rate

In general, photosynthesis can be limited by stomatal conductance, mesophyll conductance, and biochemical capacity. The relative photosynthetic limitations *l*_*s*_, *l*_*m*_, and *l*_*b*_ represent the relative importance of stomatal conductance, mesophyll conductance, and biochemical capacity, respectively, in determining the observed value of *A*_*N*_. The values of *l*_*s*_, *l*_*m*_, and *l*_*b*_ were calculated using the following equations (Grassi and Magnani, 2005):


$$l_{s}=\frac{g_{\text{tot}}/g_{\text{s}}\times \partial A_{\text{N}}/C_{\text{c}}}{g_{\text{tot}}+\partial A_{\text{N}}/C_{\text{c}}}$$

$$l_{m}=\frac{g_{\text{tot}}/g_{\text{m}}\times \partial A_{\text{N}}/C_{\text{c}}}{g_{\text{tot}}+\partial A_{\text{N}}/C_{\text{c}}}$$

$$l_{b}=\frac{g_{\text{tot}}}{g_{\text{tot}}+\partial A_{\text{N}}/C_{\text{c}}}$$

where the total CO_2_ diffusion conductance (*g*_*tot*_) was calculated as 1/*g*_*tot*_ = 1/ *g*_*s*_ +1/*g*_*m*_ (Grassi and Magnani, 2005), and the slope of the *A*_*N*_ vs. *C*_*c*_ response curve (∂*A*_*N*_/∂*C*_*c*_) was calculated according to the method of Xiong et al. (2018).

### Analysis of photosynthetic electron transport

From gas exchange parameters, the ETR for Rubisco carboxylation and oxygenation (*J*_*G*_) was calculated as follows (Zivcak et al., 2013; Walker et al., 2014):


$$J_{\text{G}}=\frac{4\times \left(A_{\text{N}}+R_{\text{d}}\right)\times \left(C_{\text{i}}+2\,\Gamma^{*}\right)}{\left(C_{\text{i}}-\Gamma^{*}\right)}$$

The alternative electron sink (*J*_*A*_) was calculated by subtracting *J*_*G*_ from ETR:


$$J_{\text{A}}=\text{ETR}-J_{\text{G}}$$

Because *J*_*G*_ represents the ETR for NADPH production, it was further divided into the two components devoted to RuBP carboxylation (*J*_*C*_) or RuBP oxygenation (*J*_*O*_) (Valentini et al., 1995):


$$J_{C}=\frac{1}{3}\times \left[J_{G}+8\times \left(A_{\text{N}}+R_{\text{d}}\right)\right]$$

$$J_{O}=\frac{2}{3}\times \left[J_{G}-4\times \left(A_{\text{N}}+R_{\text{d}}\right)\right]$$

where *J*_*C*_ indicates the rate of electron flow consumed by the Calvin-Benson cycle, and *J*_*C*_ indicates the rate of electron flow consumed by photorespiration.

### Statistical analysis

All data are displayed as mean values of five leaves from five independent plants. *T*-test was used to determine whether significant differences existed between different treatments (α = 0.05).

## Results

### Exogenous melatonin affects gas exchange during photosynthetic induction

**FIGURE 1:**
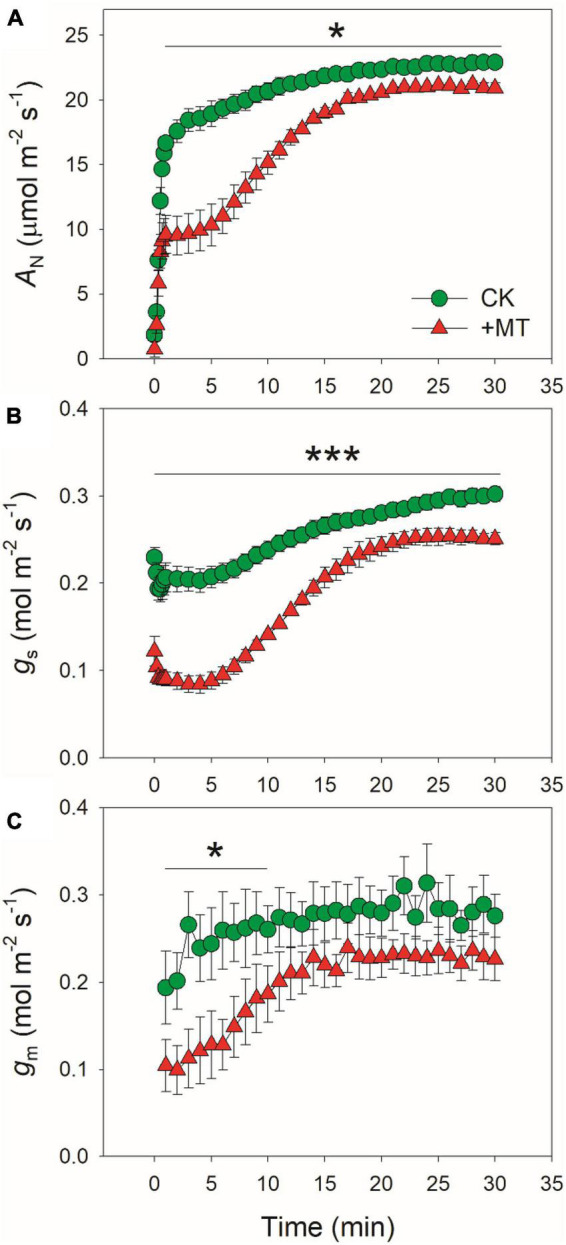
Effects of exogenous melatonin (MT, 100 μM) on the induction response of net CO_2_ assimilation rate [AN, **(A)**], stomatal conductance [gs, **(B)**], and mesophyll conductance [gm, **(C)**] after transition from 50 to 1500 μmol photons m^−2^ s^−1^. Values are means ± SE (*n* = 5). Asterisk indicates a significant difference between CK and MT-treated leaves.

The changing kinetics of *A*_*N*_, *g*_*s*_, and *g*_*m*_ during photosynthetic induction were measured by transitioning from low light (50 μmol photons m^–2^ s^–1^) to high light (1,500 μmol photons m^–2^ s^–1^) (Figure 1). The initial values of *A*_*N*_ at low light were 1.8 and 0.7 μmol photons m^–2^ s^–1^ in CK and MT-treated leaves, respectively. After this photosynthetic induction for 1 min, *A*_*N*_ rapidly increased to 16.7 μmol photons m^–2^ s^–1^ in CK leaves but just increased to 9.6 μmol photons m^–2^ s^–1^ in the MT-treated leaves (Figure 1A). After this photosynthetic induction for 5 and 10 min, *A*_*N*_ in CK leaves increased to 18.9 and 20.7 μmol photons m^–2^ s^–1^, respectively (Figure 1A). By comparison, *A*_*N*_ in MT-treated leaves increased to 10.3 and 15.2 μmol photons m^–2^ s^–1^, respectively (Figure 1A). Therefore, the induction of *A*_*N*_ after transition from low light was largely delayed by the application of exogenous melatonin. After illumination at high light for 30 min, *A*_*N*_ reached 22.9 and 20.9 μmol photons m^–2^ s^–1^ in CK and MT-treated leaves, respectively (Figure 1A), indicating that exogenous melatonin just slightly affected the steady-state AN in tobacco leaves.

Because the induction kinetics of *A*_*N*_ under fluctuating light is largely affected by *g*_*s*_ and *g*_*m*_, we further analyzed the effects of exogenous melatonin on the changing kinetics of *g*_*s*_ and *g*_*m*_ during photosynthetic induction. Under low light, *g*_*s*_ was much lower in the MT-treated leaves when compared with the CK leaves (Figure 1B). Within the first 5 min after photosynthetic induction, *g*_*s*_ in CK leaves was two-fold than that in the MT-treated leaves (Figure 1B). After photosynthetic induction for 10 min, *g*_*s*_ reached 0.24 and 0.14 mol m^–2^ s^–1^ in CK and MT-treated leaves, respectively (Figure 1B). Consistently, the transpiration rate within the first minutes after light increased was also lower in the MT-treated leaves than CK leaves (Supplementary Figure 1). Therefore, exogenous melatonin not only lowered *g*_*s*_ under low light but also delayed the stomatal opening under fluctuating light. After photosynthetic induction for 30 min, the values for *g*_*s*_ were 0.30 and 0.25 mol m^–2^ s^–1^ in CK and MT-treated leaves, respectively (Figure 1B), suggesting the slight effect of exogenous melatonin on steady-state *g*_*s*_. Similar to the performance of *g*_*s*_, the MT-treated leaves showed significantly lower *g*_*m*_ than CK leaves within the first 5 min after transition to high light (Figure 1C). However, the steady-state value of *g*_*m*_ was just slightly affected by the application of exogenous melatonin (Figure 1C).

**FIGURE 2:**
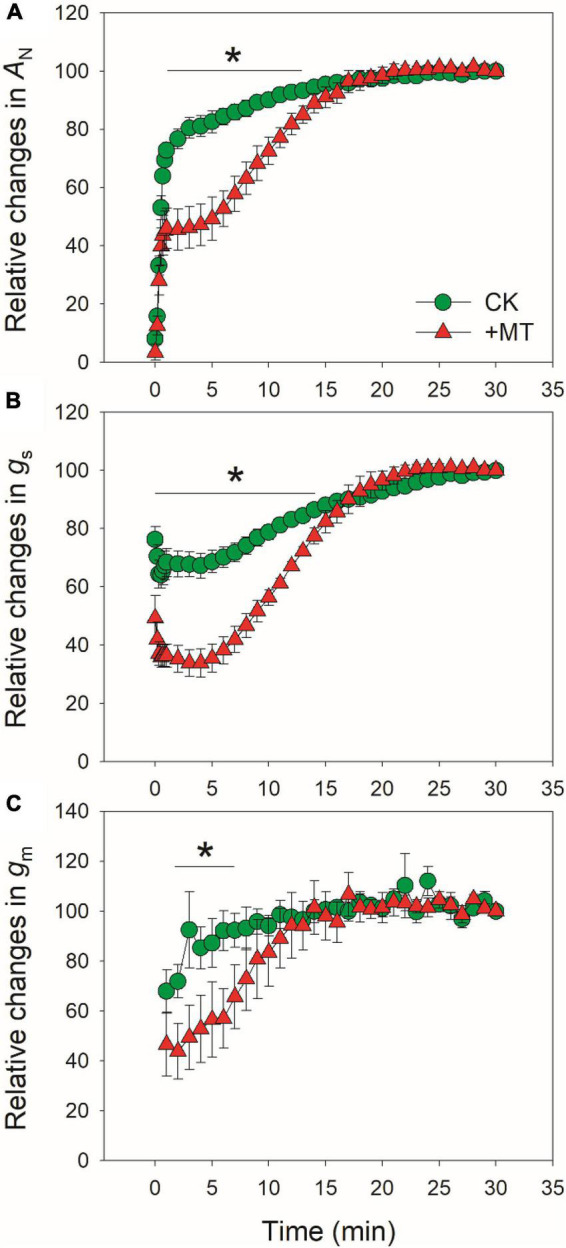
Effects of exogenous melatonin (MT, 100μM) on the relative changes in AN **(A)**, gs **(B)**, and gm **(C)** after transition from 50 to 1500 μmol photons m^−2^ s^−1^. Values are means ± SE (*n* = 5). Asterisk indicates a significant difference between CK and MT-treated leaves.

After standardization against the maximum values after 30 min photosynthetic induction at high light, the relative changes in *A*_*N*_, *g*_*s*_, and *g*_*m*_ after transition from low to high were analyzed (Figure 2). The time required to reach 80% of the maximum *A*_*N*_ was approximately 3 min in CK leaves, which was much shorter than that in the MT-treated leaves (12 min) (Figure 2A). Similarly, the time required to reach 70% of the maximum *g*_*s*_ was much lower in CK leaves (6 min) than in the MT-treated leaves (13 min) (Figure 2B). The increase in relative *g*_*m*_ was faster than *g*_*s*_ in both the CK and MT-treated leaves. However, the time required to reach 90% of the maximum *g*_*m*_ was much lower in CK leaves (3 min) than in the MT-treated leaves (12 min) (Figure 2C). These results indicated that the induction speeds of *A*_*N*_, *g*_*s*_, and *g*_*m*_ during photosynthetic induction were largely delayed upon the application of exogenous melatonin.

### Exogenous melatonin alters photosynthetic limitations during photosynthetic induction

**FIGURE 3:**
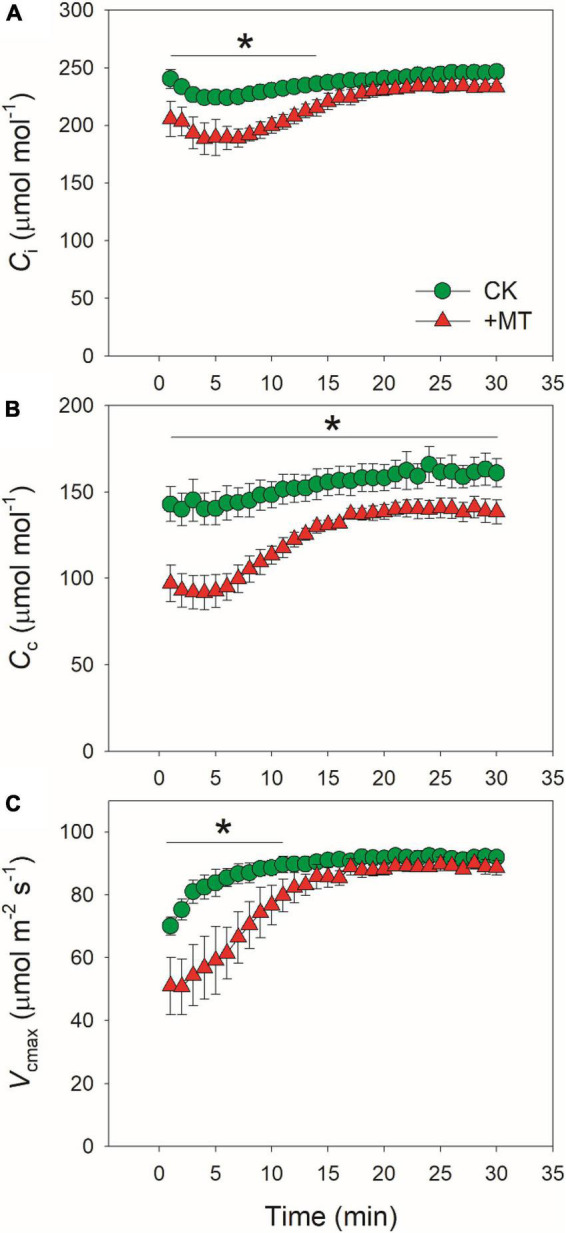
Effects of exogenous melatonin (MT, 100 μM) on the induction response of intercellular CO_2_ concentration [Ci, **(A)**], chloroplast CO_2_ concentration [Cc, **(B)**], and the maximum velocity of Rubisco for carboxylation [Vcmax, **(C)**] after transition from 50 to 1500 mol photons m^−2^ s^−1^. Values are means ± SE (*n* = 5). Asterisk indicates a significant difference between CK and MT-treated leaves.

**FIGURE 4:**
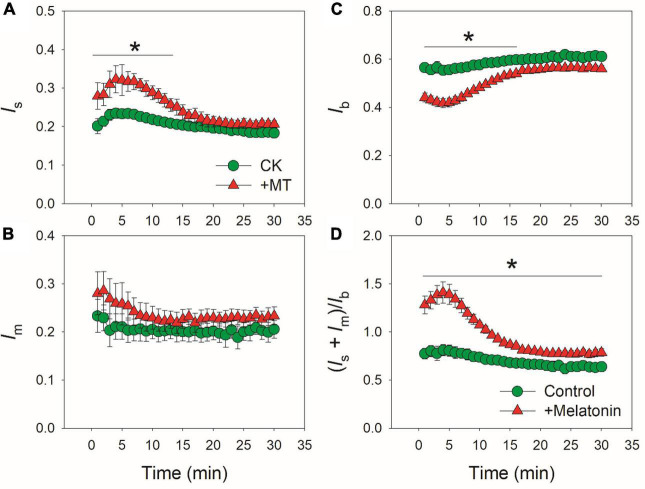
Effects of exogenous melatonin (MT, 100 μM) on the induction response of the relative limitations of gs [ls, **(A)**], gm [lm, **(B)**], biochemical factors [lb, **(C)**] and the ratio of (ls + lm)/lb **(D)** imposed to photosynthesis after transition from 50 to 1500 μmol photons m^−2^ s^−1^. Values are means ± SE (*n* = 5). Asterisk indicates a significant difference between CK and MT-treated leaves.

**FIGURE 5:**
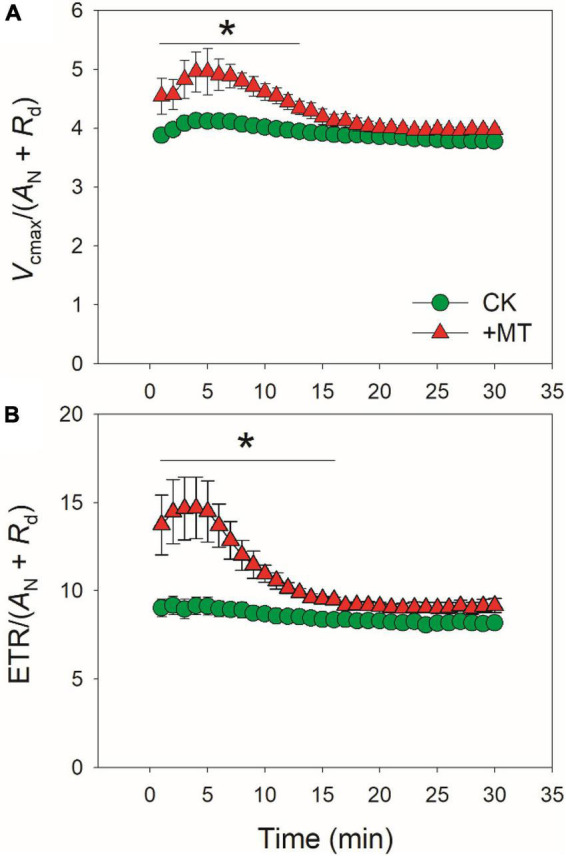
Effects of exogenous melatonin (MT, 100 μM) on the induction response of the values of Vcmax/(AN + Rd) **(A)** and ETR/(AN + Rd) **(B)** after transition from 50 to 1500 μmol photons m^−2^ s^−1^. Values are means ± SE (*n* = 5). Asterisk indicates a significant difference between CK and MT-treated leaves.

Because CO_2_ diffusion conductance determines photosynthesis through affecting intercellular (*C*_*i*_) and chloroplast CO_2_ concentration (*C*_*c*_), we calculated the response kinetics of *C*_*i*_ and *C*_*c*_ using *A*_*N*_, *g*_*s*_ and *g*_*m*_. During the initial 10 min after transition to high light, *C*_*i*_ and *C*_*c*_ were much lower in the MT-treated leaves when compared with CK leaves (Figures 3A,B). Therefore, the delayed induction kinetics of *g*_*s*_ and *g*_*m*_ in the MT-treated leaves led to the lowering of *C*_*c*_ under fluctuating light. Furthermore, the maximum velocity of Rubisco carboxylation (*V*_*cmax*_) was inhibited by the exogenous melatonin (Figure 3C), suggesting that the activation state of Rubisco was also decreased by the exogenous melatonin. During photosynthetic induction, the relative limitations of *A*_*N*_ by *g*_*s*_ (*l*_*s*_), *g*_*m*_ (*l*_*m*_), and biochemical factors (*l*_*b*_) changed slightly in CK plants (Figure 4). By comparison, *l*_*s*_ gradually decreased and *l*_*b*_ gradually increased in the MT-treated leaves. As shown in Figure 4D, the value of (*l*_*s*_ + *l*_*m*_)/*l*_*b*_ was almost lower than 1.0 in CK leaves, indicating that *l*_*b*_ was the major limiting factor of *A*_*N*_ after transition from low to high light. In contrast, the value of (*l*_*s*_ + *l*_*m*_)/*l*_*b*_ in the MT-treated leaves was higher than 1.0 within the initial 10 min of photosynthetic induction (Figure 4D), pointing out that during this period *A*_*N*_ was mainly limited by diffusional conductance. Therefore, exogenous melatonin altered the relative limitations of *A*_*N*_ during photosynthetic induction. This conclusion was further supported by the ratios of *V*_*cmax*_ and ETR to gross CO_2_ assimilation rate (*A*_*N*_ + *R*_*d*_). During photosynthetic induction, *V*_*cmax*_/(*A*_*N*_ + *R*_*d*_) and ETR/(*A*_*N*_ + *R*_*d*_) were maintained stable in CK leaves (Figure 5). However, the MT-treated leaves had higher values of *V*_*cmax*_/(*A*_*N*_ + *R*_*d*_) and ETR/(*A*_*N*_ + *R*_*d*_) during the initial 10 min of photosynthetic induction (Figure 5). After fully photosynthetic induction, the CK and MT-treated leaves showed similar values of *V*_*cmax*_/(*A*_*N*_ + *R*_*d*_) and ETR/(*A*_*N*_ + *R*_*d*_) (Figure 5). These results indicated that during photosynthetic induction the limitations of Rubisco activity and electron flow imposed to *A*_*N*_ were lowered in the MT-treated leaves compared with CK leaves.

### Exogenous melatonin enhances the capacity of alternative electron sinks

**FIGURE 6:**
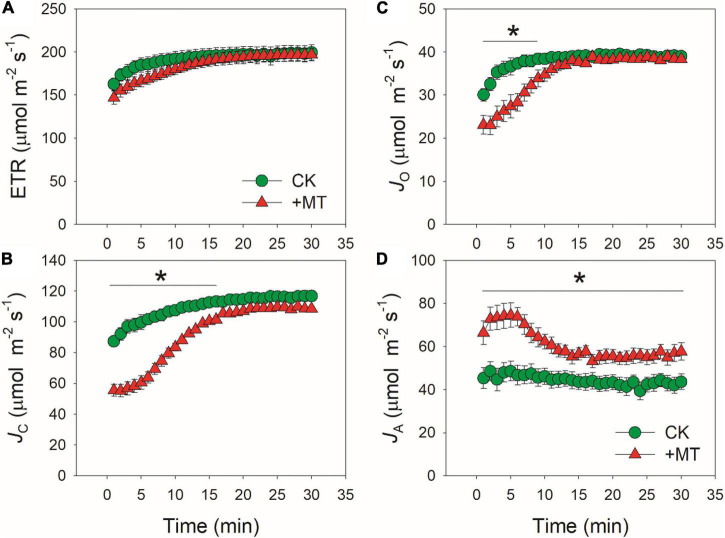
Effects of exogenous melatonin (MT, 100 μM) on the induction response of total electron transport rate (ETR) **(A)**, electron flow for Rubisco carboxylation (JC) **(B)**, electron flow for Rubisco oxygenation (JO) **(C)**, and alternative electron sinks (JA) **(D)** after transition from 50 to 1500 μmol photons m^−2^ s^−1^. Values are means ± SE (*n* = 5). Asterisk indicates a significant difference between CK and MT-treated leaves.

When CO_2_ was restricted under fluctuating light, alternative electron sinks might protect photosynthetic apparatus against photoinhibition. We analyzed the response kinetics of total PSII ETR, ETR for Rubisco carboxylation (*J*_*C*_), for Rubisco oxygenation (*J*_*O*_), and for alternative sinks (*J*_*A*_) (Figure 6). After transition from low to high light, CK and MT-treated leaves showed similar values of ETR (Figure 6A). However, the MT-treated leaves showed much lower *J*_*C*_ and *J*_*O*_ during the initial phase of photosynthetic induction (Figures 6B,C). Concomitantly, *J*_*A*_ was increased in the MT-treated leaves (Figure 6D). The maximum *J*_*A*_ in CK and the MT-treated leaves were 48.6 and 74.5 μmol electrons m^–2^ s^–1^, respectively. During photosynthetic induction, *J*_*A*_ in the MT-treated leaves was maintained at high levels in the initial 6 min but subsequently decreased gradually. By comparison, *J*_*A*_ in CK leaves was maintained stable. Therefore, the MT-treated leaves had a higher *J*_*A*_ to compensate for the restriction of *J*_*C*_ and *J*_*O*_ during the initial phase of photosynthetic induction. After fully photosynthetic induction for 30 min, CK and the MT-treated leaves showed similar ETR. However, a higher *J*_*A*_ was observed in the MT-treated leaves. These results strongly indicated that exogenous melatonin enhanced the capacity of *J*_*A*_ without altering the total ETR.

## Discussion

Recently, melatonin has been used as a plant master regulator for improving resistance to abiotic stresses (Wang et al., 2018; Arnao and Hernández-Ruiz, 2019). Generally, exogenous melatonin has the potential to modulate oxidative activity, nitrogen metabolism, secondary metabolism under these stresses, leading to the improvement of plant growth under abiotic and biotic stresses (Kaya et al., 2019, 2022; Ahammed et al., 2020; Jahan et al., 2020; Yao et al., 2021). Spraying of melatonin to the leaves is one of the most popular methods used in agriculture (Kaya et al., 2019, 2022; Jahan et al., 2020). This measure gives rise a question that whether exogenous melatonin has side effects on photosynthesis on healthy leaves. Furthermore, in view of evolutionally story of plants, it is surprising that why melatonin is not highly expressed in wild plants to enhance their resistance to environmental stresses. A possible explanation is that the content of melatonin in leaves should be controlled to a moderate level to avoid side effect on photosynthesis (Arnao and Hernández-Ruiz, 2015, 2019). However, the effects of exogenous melatonin on photosynthesis in higher plants have not yet been well known.

Under natural field conditions, plants usually experience fluctuations of light intensity on timescales of seconds, minutes, and hours owing to cloud, wind, and shading from upper leaves (Valladares et al., 1997; Slattery et al., 2018). In this study, we investigated the effects of exogenous melatonin on gas exchange and photosynthetic electron flow in tobacco plants grown under natural fluctuating light conditions. We found that the maximum *A*_*N*_ at 1,500 μmol photons m^–2^ s^–1^ was similar between the CK and MT-treated leaves (Figure 1A), indicating that the spraying of moderate concentration of melatonin (100 μM) to the leaves hardly affected the steady-state photosynthetic capacity in tobacco. However, exogenous melatonin strongly affected photosynthesis during the photosynthetic induction (Figure 1A). For example, after transitioning from 50 to 1500 μmol photons m^–2^ s^–1^ for 1 min, *A*_*N*_ increased to 16.7 μmol CO_2_ m^–2^ s^–1^ in CK leaves but just increased to 9.6 μmol CO_2_ m^–2^ s^–1^ in the MT-treated leaves. During prolonged illumination at high light for 10 min, *A*_*N*_ increased to 20.7 μmol CO_2_ m^–2^ s^–1^ in CK leaves but just increased to 15.2 μmol CO_2_ m^–2^ s^–1^ in the MT-treated leaves. Therefore, during the initial 10 min of photosynthetic induction, exogenous melatonin strongly decreased the photosynthetic carbon gain of tobacco leaves. Recent studies have documented that the rate of photosynthetic induction is an important factor affecting carbon gain and plant growth when plants grown under natural and artificial fluctuating light (Kaiser et al., 2020; Kimura et al., 2020; Yamori et al., 2020). Accelerated induction speed of *A*_*N*_ significantly enhanced biomass production in *Arabidopsis thaliana* and rice under fluctuating light (Kimura et al., 2020; Sakoda et al., 2020; Yamori et al., 2020). In tomato (*Lycopersicon esculentum*) plants treated with moderate salinity (80 mM NaCl), the induction speed of *A*_*N*_ was lowered, impairing plant growth and reducing biomass production under fluctuating light (Zhang et al., 2020). Therefore, spraying of exogenous melatonin to leaves might impair the plant growth of crops cultivated under natural fluctuating light conditions.

The induction speed of *A*_*N*_ can be affected by diffusional conductance (*g*_*s*_ and *g*_*m*_) and biochemical factors (*V*_*camx*_ and ETR) (Kaiser et al., 2017, 2020; Acevedo-Siaca et al., 2020; De Souza et al., 2020; Sakoda et al., 2021; Liu et al., 2022). We found that the MT-treated leaves displayed much lower *g*_*s*_ during initial 10 min of photosynthetic induction (Figure 1B), and *g*_*s*_ required more time to reach the maximum value in the MT-treated leaves compared with CK leaves (Figure 2B). Furthermore, induction speed of *g*_*m*_ was also delayed in the MT-treated leaves (Figures 1C, 2C). Such lowering of *g*_*s*_ and *g*_*m*_ decreased *C*_*i*_ and *C*_*c*_ during the initial phase of photosynthetic induction (Figure 3). Although the induction speed of *V*_*cmax*_ was lowered by exogenous melatonin (Figure 3C), the MT-treated leaves showed higher values of *V*_*cmax*_/(*A*_*N*_ + *R*_*d*_) during the initial phase of photosynthetic induction (Figure 5A), suggesting that exogenous melatonin did not increase the limitation of *V*_*cmax*_ imposed to photosynthesis. Similarly, the MT-treated leaves showed higher values of ETR/(*A*_*N*_ + *R*_*d*_) during the initial phase after transition to high light (Figure 5B), indicating that the limitation of ETR imposed to photosynthesis was decreased in the MT-leaves. After quantitative analysis of relative photosynthetic limitations, we found that during the initial 10 min of photosynthetic induction, *A*_*N*_ was mainly limited by diffusional conductance in the WT-treated leaves but was mainly limited by biochemical factors in CK plants (Figure 4). This altered relative photosynthetic limitation by exogenous melatonin was largely caused by the increased limitation of *g*_*s*_ imposed on *A*_*N*_. Therefore, the inhibition effect of exogenous melatonin on *A*_*N*_ during photosynthetic induction was primarily caused by the decreased induction speed of *g*_*s*_.

Previous studies have reported that exogenous melatonin can affect the expression of antioxidant systems, such as SOD and APX (Kaya et al., 2019; Jahan et al., 2020; Siddiqui et al., 2020a,b). As we know, SOD and APX are two critical antioxidant enzymes participating in an important alternative electron sink, water-water cycle (Asada, 1999, 2000; Miyake, 2010). Furthermore, the inhibition of photosynthesis requires water-water cycle to dissipate excess light energy, which is essential for protecting photosynthetic apparatus against photoinhibition (Makino et al., 2002; Hirotsu et al., 2004, 2005). However, it is unclear whether exogenous melatonin can enhance the capacity of water-water cycle to favor photoprotection. We found that the MT-treated leaves displayed much higher alternative electron sinks when ETRs for Rubisco carboxylation and oxygenation were restricted during photosynthetic induction (Figure 6). This result strongly suggested the enhancement of water-water cycle in the MT-treated leaves, because most of alternative electron flow in higher plants was accounted for the electron flux to oxygen (Asada et al., 2000; Zivcak et al., 2013; Yang et al., 2020; Ferroni et al., 2021; Sun et al., 2021). Therefore, the upregulation of water-water cycle is an important reason for why exogenous MT can strengthen photoprotection when CO_2_ is restricted under environmental stresses.

Within the first seconds after light intensity abruptly increases, plants cannot build up an enough ΔpH to fine-tune PSI redox state (Huang et al., 2019a,b). The resulting PSI over-reduction induces PSI photoinhibition under fluctuating light (Suorsa et al., 2012; Yamamoto and Shikanai, 2019). Furthermore, a decreased *g*_*s*_ could aggravate the extent of PSI over-reduction under fluctuating light (Li T. Y. et al., 2021). Upon a sudden transitioning from low to high light, alternative electron sinks can rapidly consume the reducing power in PSI and thus prevents PSI over-reduction (Gerotto et al., 2016; Jokel et al., 2018; Storti et al., 2019, 2020). Recent studies have found that water-water cycle can protect PSI under fluctuating light more efficiently than cyclic electron flow (Huang et al., 2019b; Sun et al., 2020b; Yang et al., 2020). Consequently, PSI is tolerant to photoinhibition under fluctuating light in higher plants with high capacity of water-water cycle, such as in *Camellia* species (Huang et al., 2019b; Sun et al., 2020b), *Bryophyllum pinnatum* (Yang et al., 2019), *Dendrobium officinale* (Yang et al., 2020, 2021), *Vanilla planifolia* (Wang et al., 2022). Therefore, the enhancement of water-water cycle in the MT-treated leaves can facilitate PSI photoinhibition under fluctuating light. In addition, water-water cycle can dissipate excess excitation energy and helps the formation of ΔpH, both of which are critical for photoprotection for PSII especially when CO_2_ assimilation is restricted (Miyake, 2010; Yi et al., 2014; Cai et al., 2017). Because water-water cycle generates ATP without reducing NADP^+^ and thus increases the ATP/NADPH production ratio (Miyake, 2010; Huang et al., 2016), the enhancement of water-water cycle in the MT-treated leaves can regulate the energy balancing when CO_2_ fixation is restricted. Taking together, up-regulation of water-water cycle in the MT-treated leaves has important physiological functions in photosynthetic regulation under environmental stresses.

## Conclusion

Although melatonin has many positive effects on plant tolerance under environmental stresses, we here for the first time documented that the spraying of moderate melatonin content (100 μM) to healthy tobacco leaves strongly inhibited photosynthesis during photosynthetic induction. In particular, exogenous melatonin delayed the induction speed of *g*_*s*_ after transition from low to high light. Therefore, *g*_*s*_ is the primary target of the delay effect of exogenous melatonin on photosynthesis. Furthermore, we found that the capacity of water-water cycle was enhanced in the MT-treated leaves. When photosynthesis was restricted, water-water cycle facilitated photoprotection and photosynthetic regulation in the MT-treated leaves. Therefore, exogenous melatonin has large effects on gas exchange and photoprotection in plants grown under fluctuating light.

## Data availability statement

The raw data supporting the conclusions of this article will be made available by the authors, without undue reservation.

## Author contributions

Y-JY and WH designed the study. HS, X-QW, and Z-LZ performed the photosynthetic measurements. HS, Y-JY, and WH performed the data analysis. WH wrote the first draft of the manuscript, which was extensively edited by all authors.

## Supplementary material

Supplementary Figure 1 Effects of exogenous melatonin (MT, 100 μM) on the kinetics of transpiration rate after transition from 50 to 1,500 μmol photons m^–2^ s^–1^. Values are means ± SE (*n* = 5). Asterisk indicates a significant difference between CK and MT-treated leaves.
