# Supplementary figures and images for: Exogenous melatonin strongly affects dynamic photosynthesis and enhances water-water cycle in tobacco
Source: Front Plant Sci. 2022 Aug 3;13:917784. doi: 10.3389/fpls.2022.917784 (PMC9381976; doi:10.3389/fpls.2022.917784)

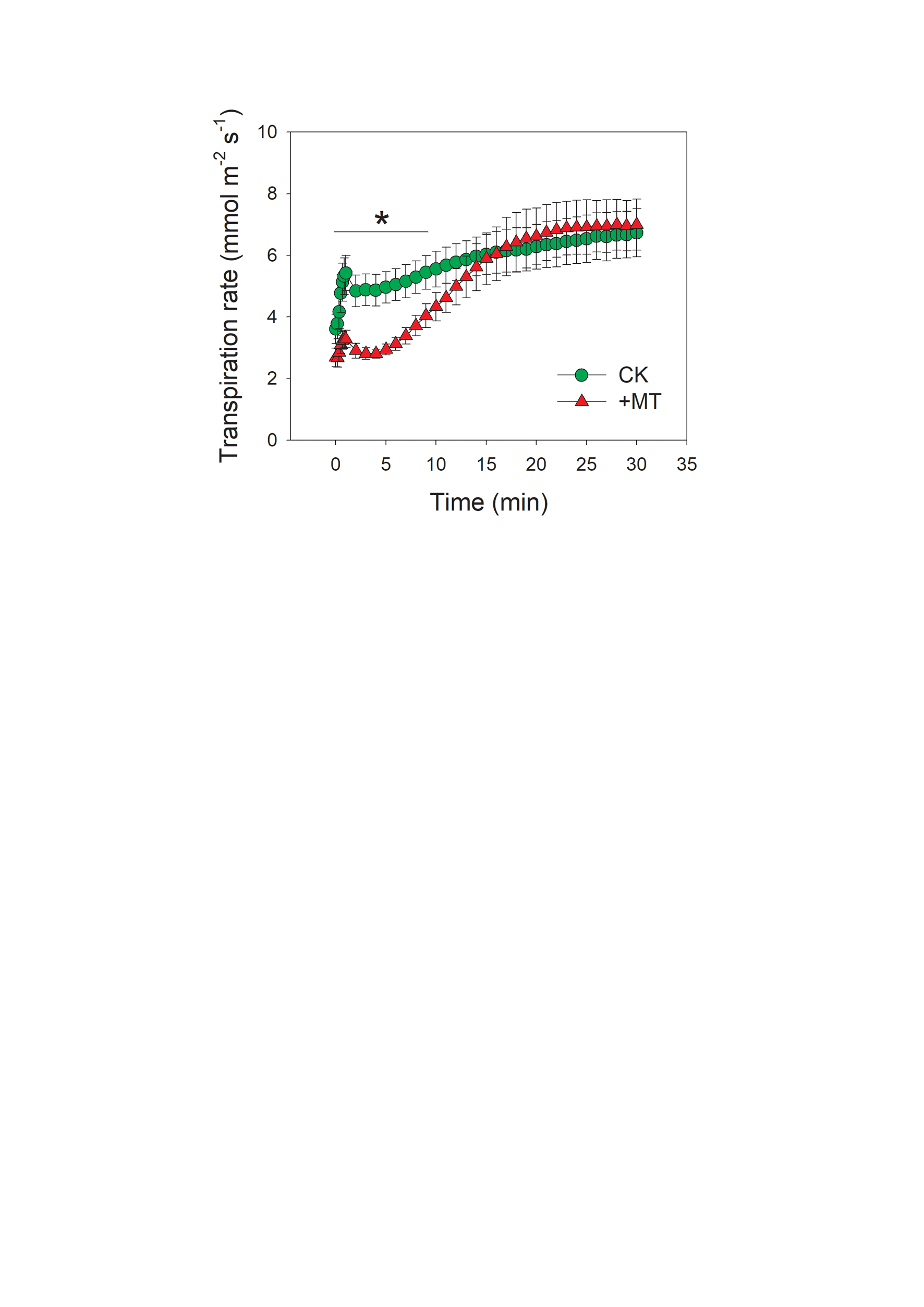

Supplement: Supplementary Figure 1 — Effects of exogenous melatonin (MT, 100 μM) on the kinetics of transpiration rate after transition from 50 to 1,500 μmol photons m–2 s–1. Values are means ± SE (n = 5). Asterisk indicates a significant difference between CK and MT-treated leaves. [file Image_1.tif]
